# Genetic evidence for functions of Chloroplast CA in *Pyropia yezoensis*: decreased CCM but increased starch accumulation

**DOI:** 10.1007/s44307-024-00019-7

**Published:** 2024-04-15

**Authors:** Baoyu Zhang, Xueying Liu, Xiujun Xie, Li Huan, Zhizhuo Shao, Zhiyan Du, Guangce Wang

**Affiliations:** 1 CAS and Shandong Province Key Laboratory of Experimental Marine Biology, Institute of Oceanology, Chinese Academy of Sciences, Qingdao, China; 2 Key Laboratory of Breeding Biotechnology and Sustainable Aquaculture, Chinese Academy of Sciences, Qingdao, China; 3 Laboratory for Marine Biology and Biotechnology, Qingdao Marine Science and Technology Center, Qingdao, China; 4 University of Chinese Academy of Sciences, Beijing, China; 5 Ocean University of China, Qingdao, China; 6 Qingdao, China; 7 Department of Molecular Biosciences and Bioengineering, University of Hawaiʻi, Mānoa, United States

**Keywords:** Chloroplastic carbonic anhydrase, Photorespiration, RNA interference, Starch accumulation, *Pyropia yezoensis*

## Abstract

In response to the changing intertidal environment, intertidal macroalgae have evolved complicated Ci utilization mechanisms. However, our knowledge regarding the CO_2_ concentrating mechanism (CCM) of macroalgae is limited. Carbonic anhydrase (CA), a key component of CCM, plays essential roles in many physiological reactions in various organisms. While many genes encode CA in the *Pyropia yezoensis* genome, the exact function of specific CA in *P. yezoensis* remains elusive. To explore the particular function of chloroplast CA in intertidal macroalgae, we produced chloroplast-localized βCA1 knockdown mutants of *P. yezoensis* through RNA interference, and *Pyβca1i* mutants (hereinafter referred to as *ca1i*) showed a notable decrease in leaf area and overall biomass, as well as decreased soluble protein and unsaturated fatty acid content under different DIC conditions. However, *ca1i* mutants showed relatively higher starch content compared to the wild-type. The activity of enzymes involved in the Calvin cycle, photorespiration, Pentose-phosphate pathway, and floridean starch synthesis of *P. yezoensis* indicated an effective starch accumulation pathway after the interference of βCA1. All results suggest that the decreased activity of PyβCA1 impaired the CCM and development of thalli of *P. yezoensis*, but stimulated starch accumulation in the cytoplasm through feedback to the photorespiration pathway and pentose phosphate pathway to replenish intermediates for the Calvin cycle. This study is the first to explore the specific function of chloroplast CA in intertidal macroalgae using genomic technology. The results provide valuable insights into the adaption mechanisms of intertidal macroalgae to their environment.

**Supplementary Information:**

The online version contains supplementary material available at 10.1007/s44307-024-00019-7.

## Introduction

On Earth, photoautotrophs utilize the photosynthetic Calvin-Benson cycle to transform inorganic carbon into organic carbon. The crucial enzyme responsible for this conversion in the Calvin cycle is Ribulose-1,5-bisphosphate carboxylase/oxygenase (Rubisco), which employs CO_2_ as a substrate. C4 higher plants employ an efficient carbon sequestration method whereby CO_2_ is initially converted into HCO_3_^−^ by carbonic anhydrase (CA), which then serves as the substrate for Phosphoenolpyruvate carboxylase (PEPC). CA is essential for C4 plants (Von Caemmerer et al. 2004). In seawater, the CO_2_ concentration is roughly 2000 times lower than in air, and diffusion is 8,000–10,000 times slower. As a result, HCO_3_^−^ is the primary inorganic carbonate in seawater (Zeebe 2011, Young et al. 2012), and marine algae (microalgae or macroalgae), utilize it as the carbonic source for growth. Therefore, studying CA is of great significance in comprehending the various forms of inorganic carbon fixation present on Earth.

Most intertidal macroalgae are economically valuable. *Pyropia yezoensis*, a representative species found in the upper intertidal zone, is an important red alga and widely cultivated in East Asia, with an annual production of about 1.8 million tons in 2017 (FAO 2019). The wild leafy thalli of *P. yezoensis* undergo emersion and submersion with the changing tides every day. Physiological research indicated that *Pyropia* could utilize atmospheric CO_2_ when emersion, while fixing HCO_3_^−^ in seawater when submerged (Zou and Gao 2002; Zhou et al. 2014; Huan et al. 2018). Thus, farmers applied the semi-floating raft method and regularly exposed *P.yezoensis* to the atmosphere during marine aquaculture, which improved the quality and yield of *P.yezoensis* (Zhou et al. 2014). Since the thalli of *P.yezoensis* are arranged in monolayer cells (Wang et al. 2004), and are surrounded by varying Ci types, achieving quick conversion between HCO_3_^−^ and CO_2_ in thalli cells of *P.yezoensis* will depend on CAs. Thus, CA is essential for *P. yezoensis*.

Diverse subtypes and distributions of CA have been found in plants and algae, including *Pyropia* (Moroney et al. 2001, Ignatova et al. 2019). To cope with the complicated intertidal environment, *Pyropia* has expanded CA genes and anti-oxidative related gene. Wang et al. (2020) suggested that 24 putative CA genes might exist in *P. yezoensis* genome, while 17 CA genes were found in *P. haitanensis* (Chen et al. 2022). Our previous studies have identified 11 CA genes with complete coding sequences, belonging to α-, β- and γ-types. The change of these PyCA genes with different [HCO_3_^−^] at the transcript level, and the intracellular localization of seven PyCAs has been defined with a fluorescent fusion protein (Zhang et al. 2020, 2022). Previous research on the DIC utilization of *P. yezoensis* was mainly based on the cellular level, and the role of PyCAs in specialized cell compartments has yet to be elucidated. Genetically transformed algal strains are necessary to explore the specific functions of PyCAs deeply.

Stable genomic transformation systems have been achieved in *P.yezoensis*, and stable mutants have been obtained based on functional genes (Zheng et al. 2021). It has been proved that genomic transformation is a powerful tool for uncovering the functions of target genes.

In this study, we focused on chloroplastic βCA1 and explored its roles in the DIC utilization of *P. yezoensis’s* leafy thalli. One of the reasons for choosing βCA1 is that it is the most abundant CA among these reported 11 PyCAs on both transcript and protein levels (Zhang et al. 2022), and the other reason is based on the importance of chloroplast for photoautotrophs. RNA interference mutants of βCA1 were obtained and tested under different bicarbonate concentrations: NC (2 mM NaHCO_3_ in seawater) and HC (8 mM NaHCO_3_). *ca1i-1* and *ca1i-2* mutants showed notable decreases in leaf area and overall biomass. Decreased βCA1 activity had adverse effects on DIC affinity and even affected the metabolism of the primary storage. This research shed light on the roles of an abundant chloroplast βCA1 in bicarbonate utilization, growth, and metabolism in the intertidal macroalga *Pyropia*, providing novel insights into the physiological mechanisms of this species.

## Results

### Target fragment insertion into *P. yezoensis* genome

**Fig. 1:**
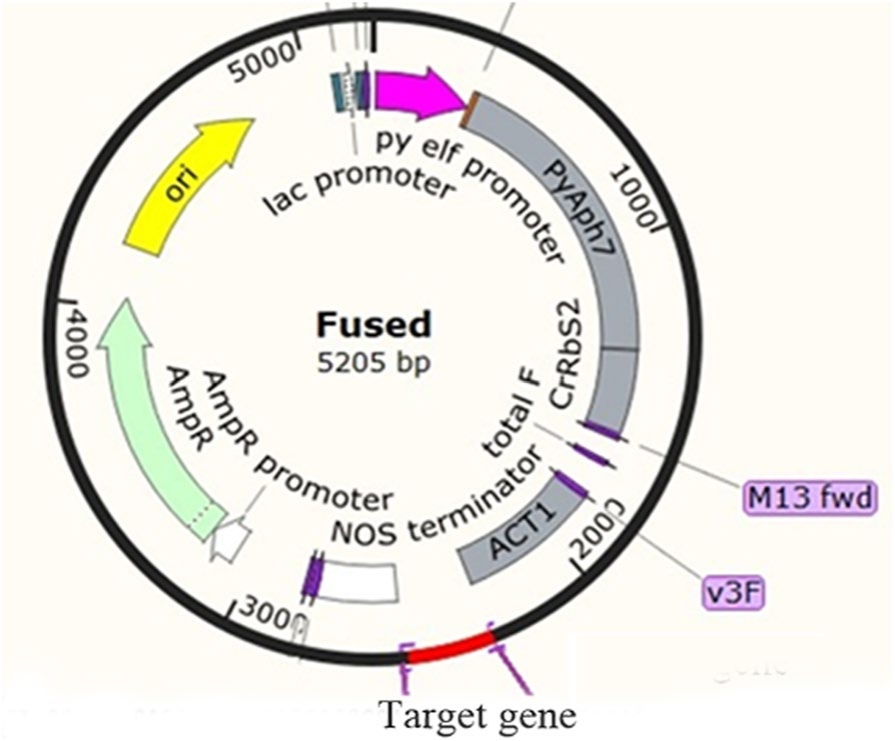
The schematic diagram of recombinant expression plasmid used in this work

Recombinant expression plasmids (Fig. 1) used for this research were transformed into leafy thalli through particle bombardment. After being subjected to hygromycin B stress for 8 weeks, five hygromycin B-resistant strains of *P. yezoensis* were isolated and named *CA1i-1*, *CA1i-2*, *CA1i-4* to *6*. To assess the stability of the expression cassettes in the *P. yezoensis* genome across generations, the mutants were re-examined after 6 months of culturing through monospores germination (Supplemental Fig. 1). The results indicated that the target fragment was stably inserted into the *P. yezoensis* genome and could be transferred through reproduction.

### The change of RNA and protein levels of *CA1i* mutants

**Fig. 2:**
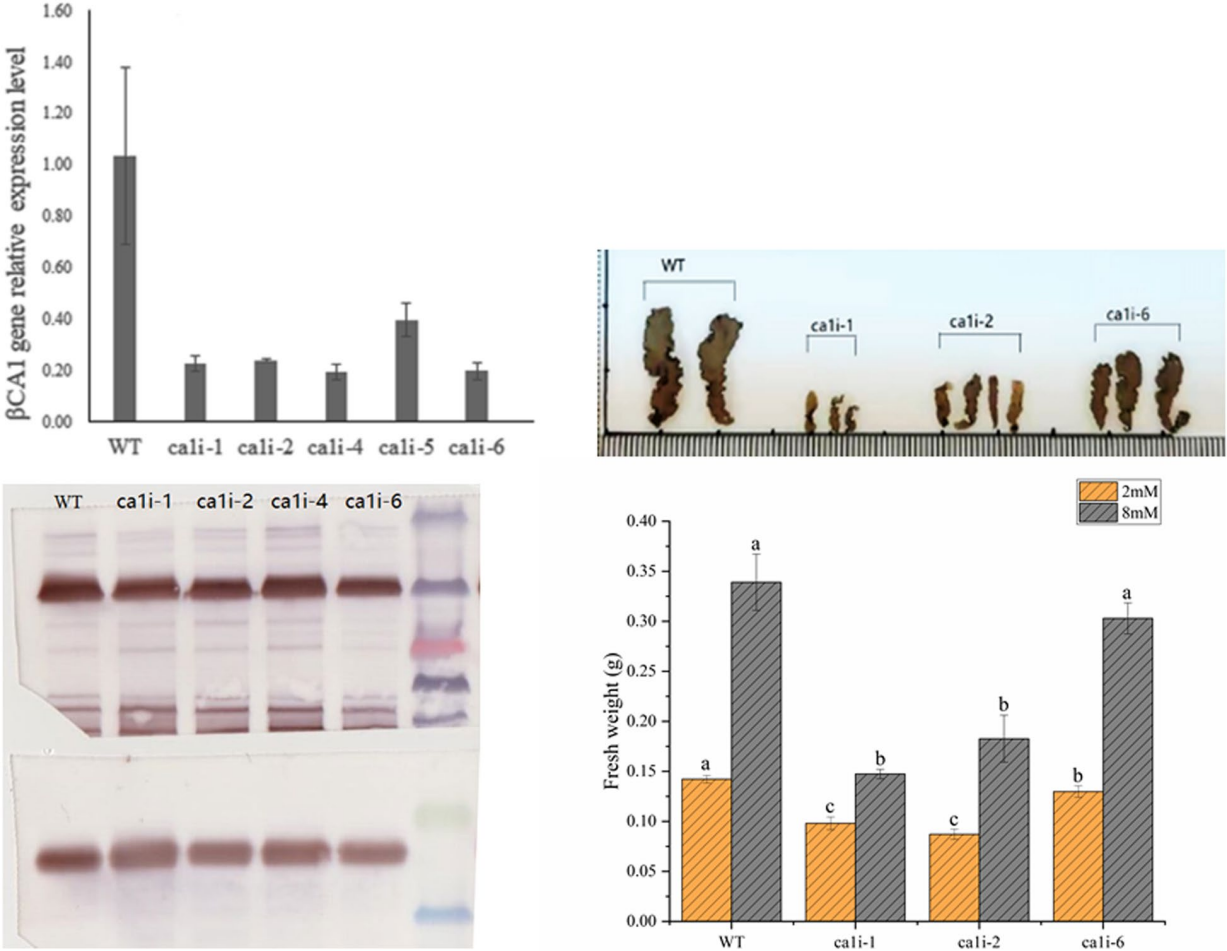
Changes in RNA and protein level of PyβCA1 among *ca1i* mutants and wild type (WT) lines, and phenotypes of *ca1i* mutants and WT under the same culture conditions. (**A**) Change in RNA level of *PyβCA1* between *ca1i* mutants and WT. (**B**) Western blot analysis of PyβCA1 in WT and *ca1i* mutants. Rubisco large-subunit (RbcL) was used as the loading control. (**C**) Phenotype of *ca1i* mutants and WT under the same culture conditions. (**D**) Fresh weight of thalli of *ca1i* mutants and WT under different DIC conditions after 14 days cultivation

The RNA and protein levels of the mutants and WT were detected by RT-qPCR and Western blot, respectively. The RNA level of mutants and WT showed a significant difference, with the *βCA1* gene expression in *ca1i* lines decreasing approximately fivefold compared to WT (Fig. 2a). An immunoblot analysis of total proteins extracts, using an anti-CA1 monoclonal antibody and a RbcL polyclonal antibody, revealed a positive band at around 28 KD and 54 KD, respectively, which matched the predicted molecular weight of PyβCA1 and RbcL protein. RbcL was used as a standard protein. The protein level of βCA1 was lower in mutants compared to WT (Fig. 2b). On the other hand, immunoblot results also indicated βCA1 was abundant in the 20 μg loading total protein.

### Morphological characteristics and biomass of *CA1i* strains

Under NC condition (2 mM NaHCO_3_), the general morphological and developmental phenotypes of *ca1i-1*, *ca1i*-*2,* and *ca1i*-*6* mutants were observed. The mutants, especially *ca1i-1* and *ca1i-2*, were visibly smaller in length and width compared with WT after 20 days of growth under the same conditions. The phenotype of *ca1i-6* was similar to WT (Fig. 2c). Based on the observed differences in RNA and protein levels, as well as morphological characteristics of the mutants, *ca1i-1*, *ca1i-2,* and *ca1i-6* were selected for further investigation.

Regarding biomass, the growth of *ca1i-1* and *ca1i-2* mutants was retarded relative to WT, resulting in a 31–38% lower biomass than WT under NC, while *cali-6* lowered biomass by approximately 9%. Under HC conditions, *ca1i-1* and *ca1i-2* mutants showed a 46–56% lower biomass than WT, while *cali-6* lowered biomass by only 11% (Fig. 2d).

### Changes in pH, photosynthetic oxygen evolution (POE), and respiration rate under different DIC conditions

To investigate the physiological responses of the *ca1i* mutants to HC (8 mM NaHCO_3_), these three strains were cultured under NC and HC conditions, with WT as control.

**Fig. 3:**
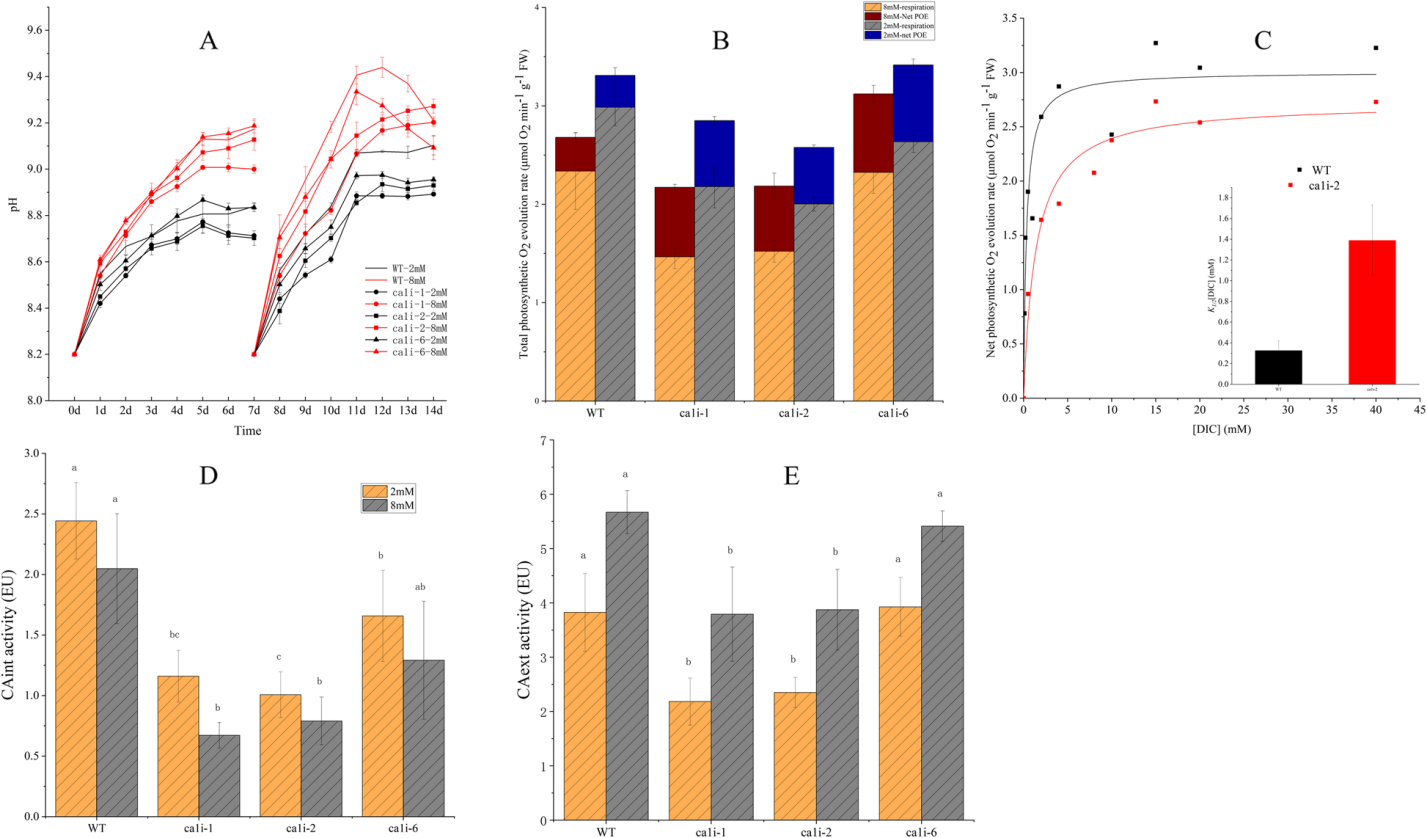
Physiological features and CA activity of *ca1i* mutants and WT under different DIC conditions. (**A**) pH change in media during culturing mutants and WT, respectively. (**B**) Photosynthetic oxygen evolution rate and respiratory rate of *ca1i* mutants and WT. (**C**-**D**) Intracellular and extracellular CA activity in mutants and WT, respectively. Data represent the mean ± standard deviation from three biological replicates. Means followed by the same lowercase letters are not significantly different at p ≤ 0.05 by one-way ANOVA and Tukey’s test. (E) Thalli of *P.yezoensis* photosynthetic DIC affinity assay and calculated [DIC] for half maximal rate = *K*_*0.5*_ [DIC]

The pH changes were similar between the *ca1i* mutants and WT under NC conditions, but the highest pH reached during 14 days of cultivation was different. The pH increased from 8.2 ± 0.02 to 8.9 ± 0.02 for the *ca1i* mutants, and to 9.1 ± 0.02 for WT. However, the pH changes in HC medium were notably faster than in NC, increasing to 9.2 ± 0.02 for *ca1i-1* and *ca1i-2* on the 14th day, whereas it increased to 9.4 ± 0.02 on the 12th day and then decreased to 9.2 ± 0.02 for WT on the 14th day, and the change of pH in *ca1i-6* was similar to WT (Fig. 3a).

The POE rate of mutants was lower than that of WT under both DIC conditions. At pH 8.2 ± 0.02, The POE rates were 1.4 and 2.2 μmol ml^−1^·gFW^−1^·min^−1^ for mutants and WT, respectively, under NC conditions. Under HC conditions, the POE rates increased to 2.0 and 3.0 μmol ml^−1^·gFW^−1^·min^−1^ for mutants and WT, respectively (Fig. 3b).

The respiration rate of these three mutant lines was all higher than in WT under both NC and HC conditions, with a rate of 0.6 μmol ml^−1^·gFW^−1^·min^−1^ for mutant lines and 0.34 for WT (Fig. 3b).

### Decreased intracellular carbonic anhydrase activity (CAint) and extracellular CA activity (CAext) compared to WT under NC and HC conditions

The primary function of CA is to catalyze the reversible interconversion of CO_2_ and HCO_3_^−^. Compared to WT, the CAint and CAext were decreased in *ca1i* mutants when cultured under NC and HC conditions (Fig. 3c). Specifically, the CAint of WT was 2.1- and 2.4-fold higher than that of *ca1i-1* and *ca1i-2*, respectively, while being 1.5-fold higher than that of *ca1i-6*. However, with increasing DIC concentration, the CAint of both mutants and WT decreased, with mutants showing a reduction of up to 40%, compared to the NC condition. In contrast, WT decreased only about 16% compared to NC conditions.

Under HC conditions, the CAext activity of both mutants and WT increased, with the CAext of WT increasing by 48%, and the CAext of *ca1i-1* and *ca1i-2* increasing by approximately 70%, compared to the NC condition. However, the CAext activity of mutants remained lower than the WT, which was 1.7–1.5 fold higher than that of *ca1i-1* and *ca1i-2* under NC and HC conditions, respectively (Fig. 3d). The CAext and CAint activity of transgenic line *ca1i-6* was similar to that of WT, with no apparent differences between them under the two DIC conditions (Fig. 3d).

### Interference of chloroplast βCA1 impaired Ci affinity and resulted in relatively higher residual [HCO_3_^−^] in the mutant medium

The half-saturation constant for DIC (*K*_*1/2*_[DIC]) represents the subject’s DIC affinity, with a higher value indicating a lower affinity for DIC. The *K*_*1/2*_[DIC] in artificial seawater-grown *ca1i* lines was approximately 4.27-fold higher than that in WT lines (Fig. 3e).

On the 7th day, the bicarbonate concentration in the medium varied differently between mutants and WT (Table 1). The residual bicarbonate concentration in the mutant medium was higher than in the WT medium, especially under HC conditions, where the divergence was more evident. After 6 days of cultivation, there was approximately 3 mM HCO_3_^−^ in the mutant medium, while it was about 1.4 mM HCO_3_^−^ for WT.

**Table 1.**
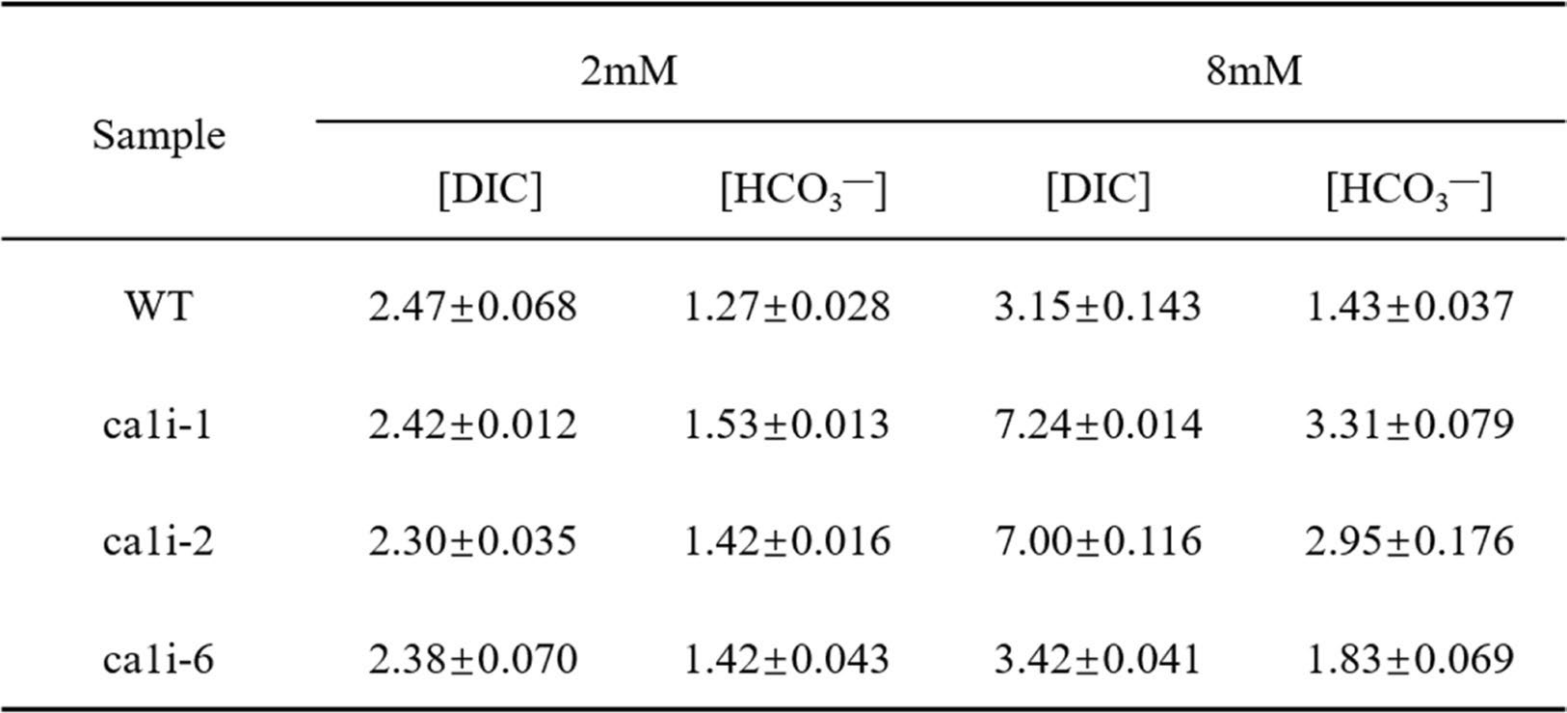
The concentration of DIC and bicarbonate in medium before and after cultivation

### The total soluble protein, fatty acid (FA), starch, and pyruvate content in mutants and WT under different DIC

**Fig. 4:**
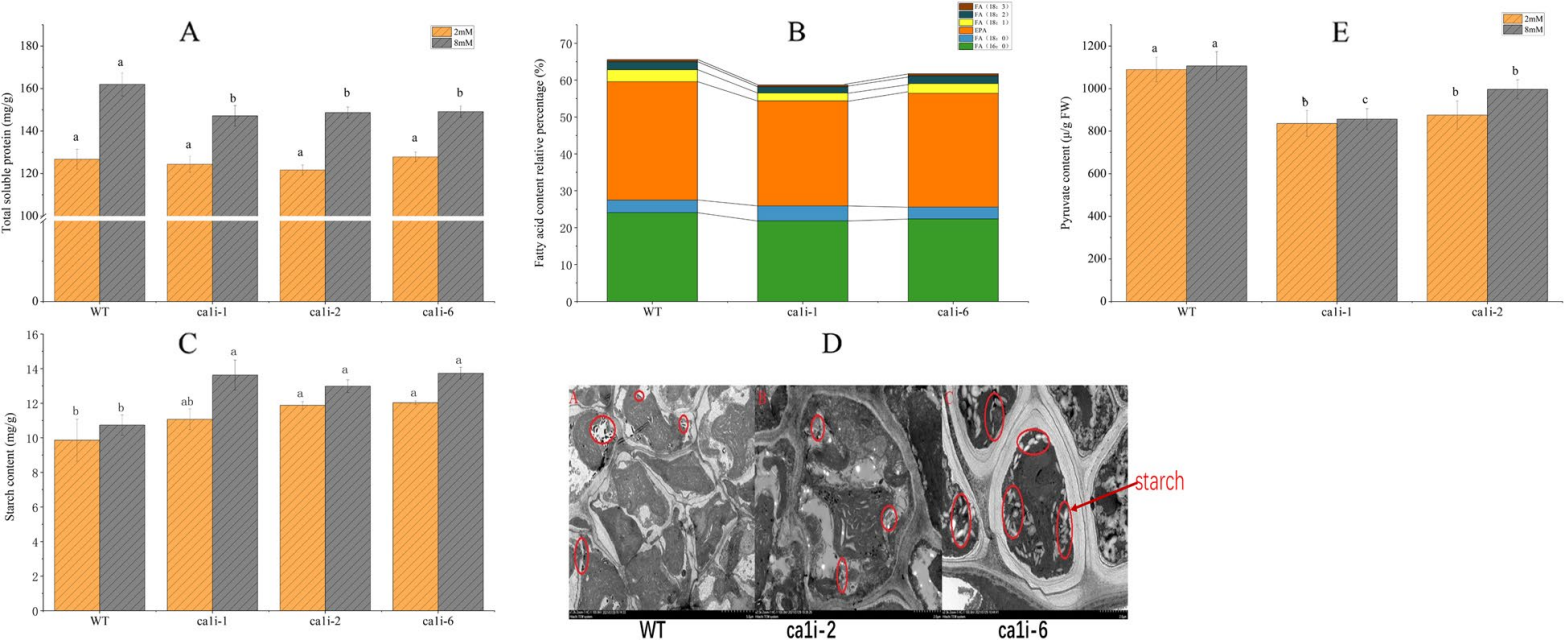
Changes in main metabolites content in mutants and WT. (**A**) Total soluble protein content among mutants and WT under different DIC conditions. (**B**) Relative content of fatty acid components in mutants and WT. (**C**) Starch content of mutants and WT under different DIC conditions. (**D**) Transmission electron microscopy images of mutants and WT thalli under normal cultivation conditions. (**E**) Pyruvate content of mutants and WT under different DIC conditions. Data represent the mean ± standard deviation from three biological replicates

*P. yezoensis* is a macroalga widely known for its high nutritional value, particularly enriched in protein, amino acids, and unsaturated fatty acid (FA). We compared the total soluble protein, FA, and starch content of *ca1i* mutants and WT under different DIC conditions after 14 days of cultivation (Fig. 4).

Under NC conditions, the total soluble protein was similar for both mutants and WT, at around 125 mg/g DW. However, under HC conditions, the total soluble protein content increased for mutants to 146 mg/g DW, while WT still had a higher content of 161.97 mg/g DW (Fig. 4a).

FA composition was then analyzed by gas chromatography and found that mutants had lower levels of C16:0, the main saturated fatty acid in *Pyropia*, compared to WT, with levels of 21.8–22.3%, and 24.1%, respectively. Additionally, mutants had lower levels of EPA, the main unsaturated fatty acid, than WT at 28.4–30% and 32.1%, respectively, under NC conditions (Fig. 4b).

The starch content for mutants and WT was 11.06–12.02 mg/g DW and 9.85 mg/g DW, respectively, under NC conditions. However, under HC conditions, the starch content increased to 12.98–13.73 mg/g DW for mutants and 10.73 mg/g DW for WT (Fig. 4c). This was further confirmed by transmission electron microscopy (TEM) images of starch accumulation in both *ca1i* mutants and WT under NC conditions (Fig. 4d).

Under the two DIC conditions, pyruvate content was significantly lower in mutants compared to WT (P < 0.05), and did not increase much with higher [HCO_3_^−^] concentration in the medium (Fig. 4e).

### Activity of enzymes involved in floridean starch synthesis, PP pathway, and photorespiration showed major divergence between the WT and *ca1i* mutants

To account for changes in pH under two DIC conditions, with pH levels not exceeding 9.0 ± 0.1 on the 3rd day, all enzyme activities were detected during cultivation on the 3rd day.

**Fig. 5:**
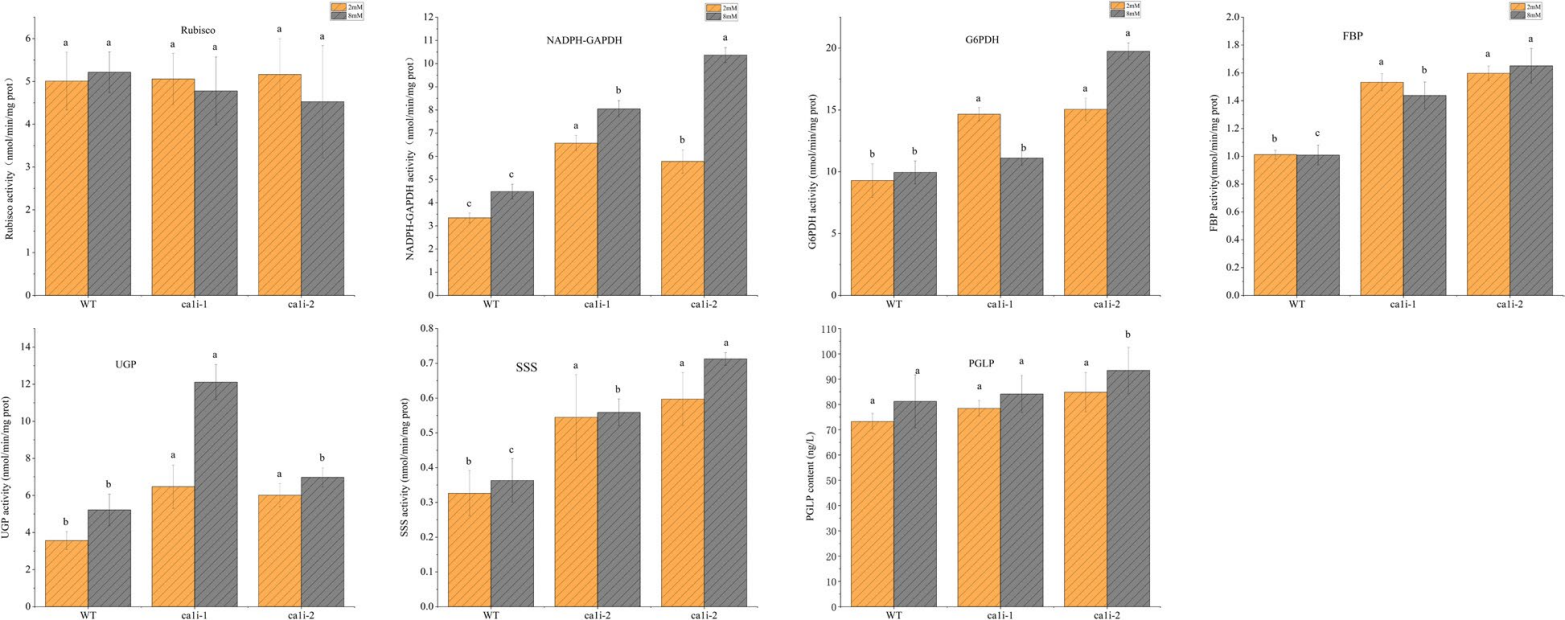
Activities of enzymes involved in Pentose-phosphate pathway, Calvin cycle, photorespiration and floridean starch synthesis. Data represent the mean ± standard deviation from three biological replicates

The Rubisco carboxylation activity was similar for both mutants and WT under NC conditions, but increased with higher [HCO_3_^−^] concentration in the WT while showing a slight decrease in mutants (Fig. 5). Glyceraldehyde-3-phosphate dehydrogenase (GAPDH) is a ubiquitous enzyme in plants, with two subtypes, one participates in glycolysis in cytosol and the other involves in the Calvin cycle in plastids. In chloroplast, GAPDH belongs to the NADPH-dependent subtype (Zaffagnini et al. 2013). NADPH-GAPDH catalyzes 1,3-Bisphosphoglycerate into 3-phosphoglyceraldehyde in the Calvin cycle and plays a central role in CO_2_ utilization. The activity of NADPH-GAPDH in mutants was significantly higher than in WT under both DIC conditions, with activity ranging from 5.7–6.5 nmol/min/mg prot to 8.0–10.3 nmol/min/mg prot with HCO_3_^−^ increase. In contrast, its activity in WT raised from 3.3 to 4.5 nmol/min/mg prot from NC to HC conditions. The activity of NADPH-GAPDH in mutants was about one fold higher than in WT (Fig. 5).

The activities of Glucose-6-phosphate dehydrogenase (G6PDH), which is involved in the Pentose-phosphate (PP) pathway, were significantly higher in *ca1i* mutants than WT under two DIC conditions, with the activity of approximately 15 nmol/min/mg prot and 9.28 nmol/min/mg prot in mutants and WT, respectively, under NC conditions. With the increase of [HCO_3_^−^] in the medium, the enzyme activity increased as well (Fig. 5).

Fructose-1, 6-bisphosphatase (FBP) catalyzes fructose-1, 6-bisphosphate conversion into fructose-6-P (Fru-6-P). The FBP activity in mutants and WT were approximately 1.5–1.6 and 1.0 nmol/min/mg prot, respectively, under NC conditions, and this enzyme activity in mutants and WT under HC conditions was similar to that under NC (Fig. 5).

UDPG pyrophosphorylase (UGP) and starch synthase (SSS) participate in the formation of carbonate storage, such as starch and floridoside, of *Pyropia* (Yu et al. 2021). The UGP enzyme activity in mutants and WT was approximately 6 and 3.55 nmol/min/mg prot under NC condition, respectively, and increased to approximately 10 and 5 nmol/min/mg prot under HC condition, for mutants and WT, respectively (Fig. 5). The change in SSS activity was similar to that of UGP activity between mutants and WT. Furthermore, the SSS activity in *ca1i-2* was about onefold higher than that of WT under these two DIC conditions (Fig. 5).

2-Phosphoglycolate phosphatase (PGLP) catalyzes the initial step of the photorespiratory pathway in various organisms, including yeast. The PGLP content under NC conditions was measured to be 73.19 ng L^−1^ for WT and around 80 ng L^−1^ for the mutants. However, under HC conditions, the PGLP content in the mutants increased to approximately 90 ng L^−1^. In contrast, the PGLP content in WT remained lower than that of the mutants, measuring 81.20 ng L^−1^ (Fig. 5).

## Discussion

### Chloroplast-localized abundant CAs play more important roles in photosynthesis and growth of aquatic photosynthetic species than the terrestrial C3 plants.

This study showed that the increase in CAext activity in the WT corresponded to the increase in HCO_3_^−^ concentration in the medium (Fig. 3d), indicating that *P.yezoensis* employs biophysical CCM by utilizing bicarbonate in seawater, consistent with previous reports (Zou and Gao 2002, Moulin et al. 2011, Zhang et al. 2020, 2022). Inorganic carbon concentration in seawater increased, as a result, leading to a decrease in the CAint activity of the WT *P.yezoensis* (Fig. 3c), which is a characteristic of CCM. CCM is induced by ambient CO_2_ in aquatic environments, but it is not active under higher CO_2_ (i.e., 30,000–50,000 ppm CO_2_) (Wang et al. 2015; Mackinder et al. 2017; Wei et al. 2019).

As an abundant chloroplast-localized CA (Zhang et al. 2020, Fig. 2b), decreasing CAint activity by 40–50% in *ca1i* lines compared with WT (Fig. 3c) led to a significant decrease in POE rate (Fig. 3b), lower affinity for bicarbonate (Fig. 2e), and even retarded growth (Fig. 2c and 2d). PyβCA1 overexpression mutants had been obtained, however, the phenotype of most mutants was similar to *ca1i* mutants (data not shown). We primarily suppose that co-suppression might occur in *βCA1* gene,which was also observed in *PyLHCI* gene in *P.yezoensis* (Zheng et al. 2021). Because the formation mechanism of co-suppression in *P. yezoensis* is yet to be elusive, thus we did not choose these co-suppression mutants as control in this work.

Highly abundant chloroplast-localized CAs might play essential roles in algae. Similar results were also observed in some microalgae such as *C. reinhardtii*, *P. tricornutum,* and *Nannochloropsis oceanica* (Wei et al. 2017; Gee and Niyogi 2017). The decrease in the thylakoid lumenal CA (CAH3) activity in *C. reinhardtii* severely impaired photosynthesis and normal physiological development, particularly under ambient CO_2_ (Karlsson et al. 1998, Markelova et al. 2009). Another abundant β-CA in *C. reinhardtii*, identified initially as low CO_2_—inducible B (LCIB), was dispersed throughout the stroma under ambient CO_2_ (Yamano et al. 2021), and played critical roles in the HCO_3_^−^ uptake; its deletion also affected normal growth under ambient CO_2_ (Adler et al. 2022).

In contrast to microalgae and macroalgae, C3 plants require minimal CA activity (Badger and Price 1994). For instance, deleting two stromal CAs in tobacco showed no effect on photosynthesis (Hines et al. 2021). This phenomenon was also observed in *Zea mays* double CAs antisense mutants (Crawford and Cousins 2022) and *Arabidopsis* deficient α-CA2 mutants (Zhurikova et al. 2016). All of these studies indicate that the activity of chloroplast-localized CAs, especially those that are highly abundant, play more crucial roles in the photosynthesis and growth of aquatic algae than in terrestrial C3 plants.

### The interference of βCA1 impaired the CCM, which decreased the assimilation of bicarbonate and reduced the content of total soluble protein and FA, but triggered a feedback mechanism that led to the accumulation of floridean starch.

The relatively high residual bicarbonate concentration in *ca1i* mutants medium under HC conditions (Table 1), and the increased *K*_*1/2*_[DIC] (Fig. 2e) indicate that the lower intracellular bicarbonate concentration in mutant lines, particularly under HC conditions, may have affected their metabolic pathways. Besides being catalyzed by CA and turning into CO_2_, HCO_3_^−^ also serves as a substrate in various pathways, such as amino acid metabolism, fatty acid synthesis and elongation, and leucine catabolism (Bauwe and Chollet 1986, Nikolau et al. 2003). Pyruvate is a key intermediate in several metabolic pathways, such as gluconeogenesis and the TCA cycle, and is essential for the synthesis of FA and protein (Nikolau et al. 2003). The synthesis of malonyl CoA and long-chain fatty acid primarily occurs in plastids (Raven 1995, Brawley et al. 2017). The relatively lower pyruvate content in mutants (Fig. 4e) led to lower FA and total soluble protein levels in mutants (Fig. 4a, 4b). However, high crude protein and unsaturated FA content in *Porphyra* (later renamed as *Pyropia*) are one of the reasons for its popularity (Fleurence 1999, Noda 1993, Blouin et al. 2006).

Rubisco is a key component of the Calvin cycle and can play carboxylation or oxygenation, depending on the ratio of CO_2_:O_2_ around it. One of the intermediates in Rubisco carboxylation is triose phosphate, which comprises glyceraldehyde-3-phosphate (G3P) and dihydroxyacetone phosphate (DHAP). These compounds also serve as substrates for starch synthesis in plants and algae. Floridean starch, floridoside, and mannitol are the main storage carbohydrates of most red algae (Rioux and Turgeon 2015, Martinez-Garcia and Maarel 2016); furthermore, the red algae produce granulated floridean starch in the cytoplasm, which is different from green algae and higher plants that store starch in the chloroplast (Viola et al. 2001; Bates et al. 2013). Fünfgeld and colleagues recently proved that ADPG pyrophosphorylase (AGP) in the chloroplasts of *Arabidopsis* was needed for starch synthesis in plastid, but not SSS in the cytosol (Fünfgeld et al. 2022). In contrast to higher plants, it is UDPG pyrophosphorylase (UGP), SSS, branching enzyme (BE), and isoamylase that contribute to the formation of carbonate storages in green and red algae (Patron and Keeling 2005; Yu et al. 2021).

Although there is no significant difference in Rubisco carboxylation between WT and mutants under two DIC conditions, mutants showed a slightly lower Rubisco carboxylation activity under HC conditions (Fig. 5a). However, the starch content in mutants was higher than in WT under NC and HC conditions (Fig. 4c). This suggests that there may be another pathway for starch accumulation or other ways to replenish the intermediates of carbohydrate storage formation. The higher NADPH-GAPDH activity in mutants (Fig. 5b) indicates that there is ample substrate, 3-PGA, for its reaction in the Calvin cycle. In addition to the pathway from Rubisco carboxylation, photorespiration from Rubisco oxygenation may also replenish 3-PGA, which coincided with relatively higher PGLP content in mutants (Fig. 5g). PGLP is a key enzyme in photorespiration and catalyzes the first reaction photorespiration C2 cycle. (Flügel et al. 2017) proved that the change of PGLP activity in photorespiration affects the content of 2-phosphoglycolate, and then affects photosynthesis and starch accumulation in *Arabidopsis*. PGLP1 overexpression lines showed significantly higher starch content than the WT, while antisense plants showed lower starch content when they were exposed to normal or low CO_2_. PGLP is also indispensable in C4 plants to maintain carbon assimilation and allocation (Levey et al. 2019).

Triose phosphate can enter the cytoplasm through chloroplast membranes and serve as a substrate for starch and carbonate storage synthesis (Viola et al.^2001^). The enzymes FBP, UGP, and SSS play key roles in this process, which results in the production of starch and floridoside. In the mutants, the higher activity of these enzymes ( as shown in Fig. 5d, 5e, 5f) supports the higher starch content compared to the WT. However, the outflow of triose phosphate from the chloroplast could lead to a decrease in the supply of RuBP, a key substrate for Rubisco. The higher activity of G6PDH in mutants (Fig. 5c) suggests that it replenishes RuBP.

**Fig. 6:**
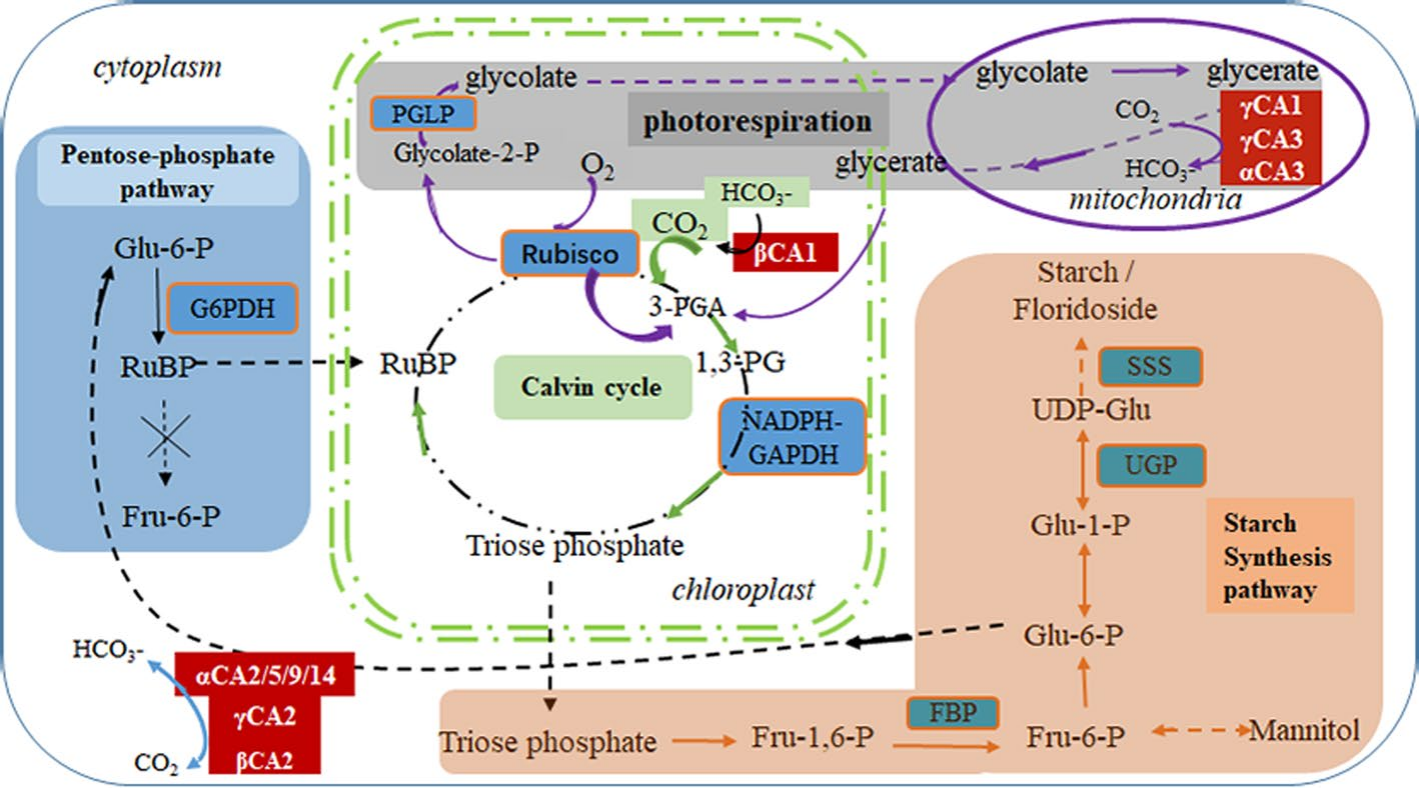
Schematic diagram of pathways which contribute to floridean starch synthesis in *P.yezoensis*, after interference of chloroplast-localized βCA1 Supplemental Fig. 1. Electrophoresis pattern of the partial fragment of *PyβCA1* amplified with the P7-βCA1i F / P7-βCA1i R primers, along with the corresponding sequencing results

In summary, the decrease of βCA1 activity impaired CCM, development, and total soluble protein and fatty acid content, but stimulated starch accumulation in the cytoplasm by feeding back such pathways as photorespiration and PP pathway to replenish intermediates for the Calvin cycle (Fig. 6). Although the mutant lines had a relatively lower [HCO_3_^−^], the higher catalytic activity of CA did not result in an obvious decrease of Rubisco carboxylation.

Our previous work found a large number of CA isoforms in *P. yezoensis* (Zhang et al. 2020), but the details about these genes encoding CA have been relatively less. Only 10 PyCAs have been defined intracellular localization through experiments until now, which were demonstrated in Fig. 6. Wang and her colleagues (2020) predicted that five α-CAs and two βCAs were targeted to the plastids of *P.yezoensis*, but this needs to be determined by experimental study. Shao et al. proved that overexpression of *PyγCA1* (renamed as *PyγCAL1*) could improve the resistance of *P. yezoensis* to high light by efficiently capturing the CO_2_ from photorespiration (Shao et al. 2022). CAs in conchocelis (one lifecycle stage of *P. yezoensis*) promote the release of HCO_3_^−^ from the shell to provide conchocelis a source of Ci (Wang et al. 2020). Our findings indicate that if there are other CAs in the chloroplast of *P. yezoensis*, the activity of other chloroplast PyCAs cannot fully compensate for the decreased activity of βCA1 during development. In addition, intertidal seaweed may employ other pathways to cope with the decrease in chloroplast-localized CA.

## Conclusions

In this study, the specific functions of highly abundant βCA1 in the chloroplast of intertidal macroalga *P. yezoensis* were studied through genetic technology. The interference of βCA1 caused retarded growth, photosynthetic O_2_ evolution, and affinity for DIC. However, antisense mutants showed similar Rubisco carboxylation and relatively higher starch content than the WT. Physiological and biochemical results suggest that chloroplast-located βCA1 is an essential component of the CCM of *P. yezoensis*. The decrease of βCA1 feedbacks the photorespiration and pentose phosphorate pathway in the leafy thalli of *P. yezoensis*.

## Materials and Methods

### Algal materials and different DIC settings

Wild-type leafy thalli of *P. yezoensis* were generated from sporophytes of this species, stored in the algae collection at the Institute of Oceanology, Chinese Academy of Sciences, Qingdao, China. The thalli were cultured in Provasoli’s enriched seawater medium at 16 ± 1 °C under 12-h photoperiod at 30 μmol·m^2^·s^−1^.

To test the reaction of leafy thalli to different [DIC], artificial seawater with different concentrations of NaHCO_3_ was used, where 2 mM and 8 mM NaHCO_3_ represented NC and HC conditions, respectively.

### Expression plasmid construction and algal transformation

Genetic transformation of *P. yezoensis* was carried out as previously described (Zheng et al. 2021) with slight modifications. The expression plasmid pEA7-PyAct1::PyCFP was kindly provided by Prof. Toshike Uji from Kokkaido University (Hirata et al. 2014). To create the *PyβCA1* RNAi expression cassette, *PyCFP* gene in this plasmid was replaced with 220 bp reverse complementary coding region of *PyβCA1* gene. This region corresponded to the partial putative active domain and amplified with the following primers, P7-βCA1i F: 5’-gggaaattcgagctcGCTGACGCACATCCGTGATG-3’, P7-βCA1i R: 5’- gtcaccttcgccaccCAGGTCACGGATGAGGCCAT-3’. The target sequence and vector via In-Fusion cloning technique, and the plasmid pEA7-PyAct1::PyβCA1i were finally produced according to the schematic diagram in Fig. 1. Particle reparation and bombardment were performed according to previous reports (Zheng et al. 2021, Shao et al. 2022). For each shooting, 10μL of the suspension was used, and the protocol of particle bombardment was the same as that described in (Zheng et al. 2021).

### Genomic PCR, qRT-PCR, and immunodetection of PyβCA1

After an 8-week selection with hygromycin B, a single thallus was chosen and cultivated in each cell culture flask, and genomic DNA was extracted from each cell culture flask with Plant genomic DNA kits (Tiangen BioTech., Beijing, China). Genomic PCR (gPCR) was conducted with the primer pairs, V3F: 5’-CGCATAAGCTTCCGCTCGA-3’ and V3R: 5’-CTGAGAGTGCACCATAAATTCCC-3’ to test the insertion of the fragment (including partial *PyAct1* promoter sequence, *PyβCA1i* interference region, and partial the *NOS* terminator sequence) into the genome of *P. yezoensis*.

To examine the expression level of the transformants, quantitative real-time PCR (qRT-PCR) was performed with PyGAPDH as the interference genes, according to Zhang et al. (2022). Total RNA was extracted using the Plant Total RNA extraction Kits (Tiangen BioTech) from both WT and mutants, and 1 μg total RNA was used to inversely transcribed to cDNA (Promega Biotech, Madison, WI, USA), which was subsequently used as templates. The protocol for qRT-PCR was similar to that described in Zhang et al. (2020), and the primer pairs for qRT-PCR were as follows: F: 5’-CCA GTC TGC TTG GAACGACG-3’, R: 5’-TAGACCGAATCGATGCCCTC-3’.

Western blotting was performed following the protocol described in Zhang et al. (2022). Specifically, 20 μg of total soluble protein from WT and mutants were loaded per lane and separated in 12% SDS-PAGE. Monoclonal primary antibody against PyβCA1 was produced by Youke Biotech. Co. (Shanghai, China), while polyclonal antibodies against RbcL for plants and algae were obtained from Agrisera (Product No. AS03037). Anti- PyβCA1 or anti-RbcL secondary antibodies were either anti-mouse IgG, HRP conjugated, or anti-rabbit IgG, HRP conjugated (GenStar).

### pH change and fresh weight under different DIC conditions

The pH was monitored daily using a pH meter (Sartorius, Beijing, China) and the medium was changed weekly. On the 14th day, leafy thalli were harvested through a cell filter (Ф100 μm), and redundant water was removed through filter paper. The samples were then weighed and frozen at -80℃ for further use.

### DIC affinity curves and half-saturation determination

DIC-dependent O_2_ evolution was measured using a Clark-type Oxygen Electrode (Hansatech Instruments, UK) at 50 μmol·m^2^·s^−1^ and 15 ± 0.1℃. Leafy thalli were cut into small pieces (ca. 2 mm × 2 mm) and incubated in artificial seawater (ASW) without NaHCO_3_ under normal cultivation conditions for 24 h to minimize the effect of cutting damage on photosynthesis. Approximately 10 mg of the sample was transferred to a chamber containing 2 mL DIC-free ASW, and reaction media were magnetically stirred. NaHCO_3_ was injected into the chamber when no further O_2_ evolved, creating various DIC concentrations by adding aliquots of NaHCO_3_ stock solution into the media. O_2_ evolution was recorded within 10 min after the addition of NaHCO_3_. Photosynthesis curves were processed using a Python (http://www.python.org/) script that utilized the scipy ‘curve_fit’ function to fit a Michaelis–Menten equation to the data and solve for Vmax and* K*_*1/2*_[DIC].

### Photosynthetic oxygen evolution rate (POE) and respiration rate, total DIC and HCO_3_^−^ concentration under different DIC conditions

The net POE and respiration rate of WT and mutants were also determined by a Clark-type oxygen electrode as described above, and the respiration rate was measured for 10 min after dark.

Total alkalinity of ASW was determined by titration of seawater at 32 (PSU) and 15 ± 0.1℃ using the method described by Millero et al. (1993), and total [DIC] in the medium after 6 days of cultivation was measured using LI-7000 CO_2_/H_2_O analyzer (LI-COR, USA). All data, including temperature, alkalinity, pH, and total [DIC], were input into CO2SYS (Pierrot et al. 2006) and used to calculate HCO_3_^−^ concentration.

### Measurement of total soluble protein, starch, pyruvate, PGLP content, and free fatty acid (FA) analysis

The leafy thalli were subjected to freeze-drying and used to measure the total soluble protein and starch content. Approximately 30 mg of dried powder was extracted according to the Plant total protein kit protocol ( Solarbio Sci & Tech Co. Ltd, Beijing). Similarly, 30 mg of dried powder was conducted according to the protocol of the Total Starch Content Kit (Geruisi-bio. Co. Suzhou, China). and the absorbance was measured at 510 nm. The total starch content was calculated by multiplying the glucose content by the conversion coefficient of 0.9.

The pyruvate content in the leafy thalli of the WT and mutants was quantified using the Plant Pyruvic Acid Content Kit (Grace Biotech, China) according to the provided protocol. Pyruvic acid reacts with 2,4-dinitrophenylhydrazine to form 2,4-dinitrophenylhydrazone, which exhibits a brownish-red color in an alkaline solution. The absorbance value at 520 nm was measured to determine the pyruvic acid content. Similarly, the PGLP content in the leafy thalli of WT and mutants was assessed using the PGLP Content ELISA Kit (Shanghai Enzyme-Linked Biotech. Co. Ltd., China) following the instructions provided. The absorbance value at 520 nm was measured for quantification purposes.

The total lipid was extracted from 30 mg freeze-dried leafy thalli using a chloroform–methanol method described by Wu et al. (2015). The crude lipid samples were dried with N_2_ flow until a constant weight was obtained. The fatty acid compositions of different DIC-cultivated mutants and WT leafy thalli were determined by gas chromatography.

### Enzyme assay

CA activity was carried out according to the methods of Fernández et al. (2018) with slight modifications. For external CA (CAext) activity, the time required for the pH to drop from 8.4 to 7.9 was recorded at 4–5℃ using a chamber containing 5 mL of cold Tris–Cl buffer (50 mM tris–cl, 5 mM EDTA, 2 mM DTT, pH 8.5 ± 0.01, 4℃). Approximately 0.01 g of fresh leafy thalli was carefully prepared by removing any excess water from the surface with filter paper. Next, 2 mL of cold (4℃) CO_2_-saturated MilliQ water (18.3 MΩ cm) was added to the chamber to initiate the reaction. Enzyme activity was expressed in enzyme unit (EU), calculated using the formula: EU = 10 × (T0-T)/T, where T0 and T represent the time in seconds for pH drop without and with tissue, respectively. To determine total CA activity, both internal and external, crude extracts were obtained by grinding 50 mg of the sample with sample buffer used for extracellular activity determination. The resulting 150 ul homogenate was added to 4.85 mL Tris–Cl buffer, and the total CA activity was measured in the same manner as for CAext. Intracellular CA (CAint) was calculated by subtracting CAext activities from total CA activity.

The enzymatic activities of Rubisco, NADPH-glyceraldehyde-3-phosphate dehydrogenase (GAPDH), glucose-6-phosphate dehydrogenase (G6PDH), Fructose-1, 6-bisphosphatase (FBP), starch synthase (SSS), UDPG pyrophosphorylase (UGP) were measured using quantification kits (Grace Biotech, China) following the user’s manual. In the protocol, about 100 mg of fresh algal materials were ground on ice, and suspended with extraction buffer. After concentration, supernatants were incubated on ice for further enzyme assays. Enzyme activities were determined using microplate readers (Infinite M1000 pro, Sweden) by measuring the change in absorbance at 340 nm. The total protein concentration of supernatants was measured by absorbance at 562 nm with the BCA protein assay kit (GenStar). The enzyme activity unit was defined as nmol NAD(P)H oxidation or NAD(P) + reduced per minute per milligram total protein. The SSS activity was determined by measuring the change in absorbance at 450 nm according to the kit protocol.

### Statistical analysis

For all measurements of metabolites and enzyme activities, one-way ANOVAs with post-hoc Tukey test analyses were performed using IBM SPSS Statistics 23 (IBM Co., Aemonk, NY, USA). Statistical significance was set at P ≤ 0.05. The graphs were plotted using Origin2018 (OriginLab Co., Northampton, MA, USA).

## Supplementary Information

**Supplementary Material 1.**

## Data Availability

All data generated or analyzed in this study are included in this published article and its supplementary information file.
