# Supplementary figures and images for: Genetic evidence for functions of Chloroplast CA in Pyropia yezoensis: decreased CCM but increased starch accumulation
Source: Adv Biotechnol (Singap). 2024 Apr 15;2(2):16. doi: 10.1007/s44307-024-00019-7 (PMC11740840; doi:10.1007/s44307-024-00019-7)

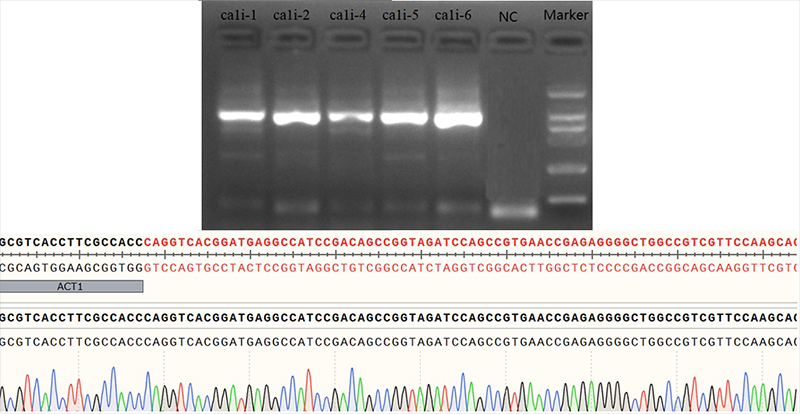

Supplement: Supplementary file 1 — Supplementary Material 1. [file 44307_2024_19_MOESM1_ESM.tif]
